# TRIM44 activates the AKT/mTOR signal pathway to induce melanoma progression by stabilizing TLR4

**DOI:** 10.1186/s13046-019-1138-7

**Published:** 2019-03-28

**Authors:** Chuan-Yuan Wei, Lu Wang, Meng-Xuan Zhu, Xin-Yi Deng, Dao-He Wang, Si-Min Zhang, Jiang-Hui Ying, Xin Yuan, Qiang Wang, Tian-Fan Xuan, An-Qi He, Fa-Zhi Qi, Jian-Ying Gu

**Affiliations:** 1 Department of Plastic Surgery, Zhongshan Hospital, Fudan University, 180 Fenglin Road, Shanghai, 200032 People’s Republic of China; 2 Department of Liver Surgery, Liver Cancer Institute, Zhongshan Hospital, Key Laboratory of Carcinogenesis and Cancer Invasion, Ministry of Education, Fudan University, Shanghai, 200032 People’s Republic of China; 3 Liver Cancer Institute, Zhongshan Hospital, Key Laboratory of Carcinogenesis and Cancer Invasion, Ministry of Education, Fudan University, Shanghai, 200032 People’s Republic of China

**Keywords:** TRIM44, Melanoma, EMT, Prognosis, TLR4, AKT/mTOR pathway

## Abstract

**Background:**

There is growing evidence that tripartite motif-containing protein 44 (TRIM44) plays crucial role in tumor development. However, the underlying mechanism of this deubiquitinating enzyme remains unclear.

**Methods:**

Large clinical samples were used to detect TRIM44 expression and its associations with clinicopathological features and prognosis. Gain- and loss-of-function experiments in cell lines and mouse xenograft models were performed to elucidate the function and underlying mechanisms of TRIM44 induced tumor progression. Co-immunoprecipitation (Co-IP) assays and mass spectrometric analyses were applied to verify the interacting proteins of TRIM44.

**Results:**

We found that TRIM44 was commonly amplified in melanoma tissues compared with paratumoral tissues. TRIM44 expression also positively correlated with more aggressive clinicopathological features, such as Breslow depth (*p* = 0.025), distant metastasis (*p* = 0.012), and TNM stage (*p* = 0.002). Importantly, we found that TRIM44 was an independent indicator of prognosis for melanoma patients. Functionally, overexpression of TRIM44 facilitated cell invasion, migration, apoptosis resistance and proliferation in vitro, and promoted lung metastasis and tumorigenic ability in vivo. Importantly, high level of TRIM44 induced melanoma cell epithelial-mesenchymal transition (EMT), which is one of the most important mechanisms for the promotion of tumor metastasis. Mechanistically, high levels of TRIM44 increased the levels of p-AKT (T308) and p-mTOR (S2448), and a specific AKT inhibitor inhibited TRIM44-induced tumor progression. Co-IP assays and mass spectrometric analyses indicated that TRIM44 overexpression induces cell EMT through activating AKT/mTOR pathway via directly binding and stabilizing TOLL-like receptor 4 (TLR4), and TLR4 interference impeded TRIM44 induced tumor progression. Moreover, we demonstrated that TRIM44 is the target of miR-26b-5p, which is significantly downregulated in melanoma tissues and may be responsible for the overexpression of TRIM44.

**Conclusions:**

TRIM44, regulated by miR-26b-5p, promotes melanoma progression by stabilizing TLR4, which then activates the AKT/mTOR pathway. TRIM44 shows promise as a prognostic predictor and a therapeutic target for melanoma patients.

**Electronic supplementary material:**

The online version of this article (10.1186/s13046-019-1138-7) contains supplementary material, which is available to authorized users.

## Introduction

There is an increasing incidence of cutaneous melanoma, which now accounts for 232,100 (1.7%) of all newly diagnosed primary malignant tumors annually. However, melanomas are indistinguishable from benign nevus and, as a result, are easily overlooked [1]. Once melanoma has spread the cancer rapidly becomes life-threatening due to its characteristics of abnormal proliferation and early metastasis. The disease causes 55,500 deaths (0.7% of all cancer deaths) every year [2]. Understanding the disease progression may help researchers identify new targeted therapies and markers for improved prognostic accuracy.

TRIM family proteins are characterized by one or two zinc-binding motifs, B-boxes, and an associated coiled-coil region [3]. Most have E3 ubiquitin ligase activities as they contain a RING-finger domain. They are involved in a variety of cellular processes, such as protein degradation, cell cycle progression, transcriptional regulation, DNA repair, and signal transduction [4, 5]. There is growing evidence that the dysregulation of TRIM family proteins leads to a range of disease pathological conditions, including cancer [6]. For example, Lv et al. shows that TRIM24 is a transcriptional co-activator in EGFR-driven glioblastoma by which H3K23ac/TRIM24 mediates EGFR stimulation of STAT3 pathway [7]. Interestingly, studies indicate that, unlike most TRIM family members, TRIM44 contains a zinc finger ubiquitin protease domain (UBP) and functions as a deubiquitinating enzyme [8–10]. For example, Urano et al. demonstrates that TRIM44 functions as a “USP-like TRIM” and regulates the deubiquitination and stabilization of E3 ligase [8]. In addition, Chen et al. finds that TRIM44 stabilizes hypoxia inducible factor-1α and stimulates multiple myeloma cell growth under hypoxia [10]. Despite this evidence, only a handful of publications link TRIM44 function with cancers and the mechanisms underlying this association are even less clear.

MicroRNAs (miRNAs) belong to a family of small non-coding RNA molecules and are generally about 20 nucleotides long [11]. The primary role of miRNAs is to regulate protein translation by binding to complementary sequences in the 3′ untranslated region (UTR) of target messenger RNAs (mRNAs), which results in mRNA translational inhibition or degradation [12, 13]. Over past decades, functional studies have determined that miRNA dysregulation is the cause of many cases of cancer, a role in which miRNAs can act as either tumor suppressors or promoters. Molecules targeting miRNAs have shown promise in preclinical development [14, 15]. For example, miR-34a, a downstream tumor suppressor miRNA of p53, antagonizes the key hallmarks of oncogenes and plays imortant roles in self-renewal, migratory potential, and chemo-resistance. As a result, it has become one of the first miRNA-based cancer therapies [16, 17]. By directly targeting TRIM4, Wang et al. reports that overexpression of miR-410 suppresses osteosarcoma proliferation, migration, invasion, and EMT [18]. However, it remains unknown whether other miRNAs bind to the 3′ UTR of TRIM44.

In this study, we explored the expression and biological functions of TRIM44 using a comprehensive investigative approach that included clinical melanoma samples along with cellular and animal models. Furthermore, we elucidated the mechanisms behind TRIM44-induced melanoma cellular EMT, as well as upstream TRIM44 regulators.

## Materials and methods

### Patients and follow-up

Twenty pairs of melanoma tissue samples with matched normal tissues were obtained from randomly selected patients seen at Zhongshan Hospital, Fudan University (*Shanghai,* China). Samples were used for Western blot and quantitative reverse-transcription polymerase chain reaction (qRT-PCR) analyses. A total of 138 paired melanoma and matched normal tissues, along with an additional 59 melanoma tissues, were used for tissue microarray (TMA) as previously described [19]. The Ethics Committee of the Zhongshan Hospital Biomedical Research Department approved this study. All patients provided written informed consent.

### Immunohistochemistry (IHC)

IHC was performed as previously described [20]. Briefly, the slides were deparaffinised in xylene and rehydrated in graded ethanol. After incubation in 0.3% H_2_O_2_, antigen retrieval was performed in citrate buffer. Subsequently, sections were incubated with the primary antibody (Additional file 1: Table S1), horseradish peroxidase (HRP)-labeled secondary antibody (Gene Tech, Shanghai, China), stained with diaminobenzidine (DAB, Gene Tech, Shanghai, China), counterstained with hematoxylin, then, dehydrated in ethanol, cleared in xylene, and cover-slipped with resin. The integrated optical density (IOD) value was assessed by Image Pro Plus software (V 6.0).

### Cell culture and transfection

Melanoma cell lines A2058, A375, A875, MV3 and M14 were purchased from the cell bank of the Chinese Academy of Sciences (*Shangh*ai, China). MV3 and M14 cells were cultured in Roswell Park Memorial Institute-1640 medium (RPMI-1640, HyClone, Logan, UT, USA); A2058, A375 and A875 were cultured in Dulbecco’s modified Eagle’s medium (DMEM, HyClone, Logan, UT, USA). All media were supplemented with 10% FBS (Gibco, Waltham, MA, USA), streptomycin sulfate (100 IU/mL) and penicillin (100 μg/mL). All cells were incubated in a humidified incubator at 37 °C with 5% CO_2_.

The pGMLV-SC5-Puromycin-EGFP-shRNA-TRIM44 lentiviral vectors (Genomeditech, Shanghai, China) were transfected into A2058 cells. The pGMLV-PE3-RFP-TRIM44 lentiviral vectors (Genomeditech, Shanghai, China) were transfected into A375 cells. The shRNA1 sequence used was GCCTTTGAAGAATTAAGAAGC and the shRNA3 sequence was GCAGAAGGCCCTTCATCTAGT. Transfection efficiency was determined by qRT-PCR and Western blot.

### Western blot and qRT-PCR

Western blot was conducted as previously described [21]. Cell lysates were obtained using RIPA buffer (Beyotime, Shanghai, China). Total protein was injected into sodium dodecyl sulfate-polyacrylamide gel electrophoresis (SDS-PAGE) and transferred to polyvinylidene difluoride (PVDF) membranes. After blocking with 5% BSA, the membranes were incubated with primary antibody (Additional file 1: Table S1), HRP-conjugated secondary antibody (Yeasen, Shanghai, China) according to the manufacturer’s directions. Bands were visualized with a chemiluminescent HRP substrate (Millipore, MA, USA) and an electrogenerated chemiluminescence (ECL) imaging system (Tanon, Shanghai, China). Densitometric analysis was performed using Adobe Photoshop CS6 (CA, USA).

qRT-PCR was performed as previously described [22]. Briefly, total RNA was extracted by TRIzol reagent (Invitrogen, Carlsbad, CA, USA). Complementary DNA was synthesized using a high capacity cDNA reverse transcription kit (Takara, Dalian, China). qRT-PCR was conducted with the Applied Biosystems 7500 Real-Time PCR System using a SYBR Green Real-Time PCR Master Mix kit (Takara, Dalian, China). Primers for qRT-PCR are shown in Additional file 2: Table S2.

### Invasion, wound healing, and CCK8 assays

For Transwell invasion assays, cells were incubated in Corning BioCoat Matrigel invasion chambers (24-well, 8-μm pore size, Corning, NY, USA). A total of 10^4^ cells were seeded on Matrigel-coated inserts in 200 μL of serum-free DEME. 600 μL DMEM with 10% FBS was added to the bottom chambers. After 48 h, cells were fixed in 4% paraformaldehyde and stained by crystal violet for 15 min. The number of cells per three randomly selected fields was counted under the microscope (Olympus, Tokyo, Japan). For wound healing assays, artificial wounds were scratched on a confluent cell monolayer by 200 μL pipette tips until cells covered 95% of the 6-well plated bottom. Then wound healing images were taken at an appropriate time. For CCK-8 assay, cells were seeded in 96-well plates (1000 cells/well) in triplicate. At various time points, groups of cells were incubated with 10 μL Cell Counting Kit-8 (CCK-8, Yeasen, Shanghai, China) for 3 h at 37 °C. The absorbance values were measured at 490 nm.

### In vivo assay

Five-week-old athymic BALB/c nude mice were purchased from the Shanghai Institute of Material Medicine and maintained in a pathogen-free environment. For the metastasis model, 2 × 10^6^ cells were injected into the mouse tail veins (*n* = 6 per group). After 4 weeks, the mice were sacrificed and lungs were obtained for histological analysis. The number of metastatic tumors in the lungs was counted. For the growth model, 2 × 10^6^ cells were injected into the backs of the mice (n = 6 per group) to generate subcutaneous tumors. Tumor size was measured every 5 days and the tumor volume was calculated as: length x width^2^ × 0.5. Thirty days after injection, the tumor specimens were surgically dissected and subjected to immonohistochemical staining. All experimental procedures were approved by the Animal Care Committee of Fudan University (Shanghai, China).

### Immunofluorescence and flow cytometric assays

For immunofluorescence staining, cells were fixed with 4% formaldehyde, permeabilized with PBS containing 0.3% Triton X-100 (Beyotime, Shanghai, China), and blocked with 5% BSA. The cells were treated with the primary antibody (Additional file 1: Table S1) and fluorescent secondary antibody (Yeasen, Shanghai, China) as recommended. Then, cells were washed with PBS, stained with 4′, 6-diamidino-2-phenylindole (DAPI, Yeasen, Shanghai, China), and evaluated by fluorescence microscopy (Leica Microsystems Imaging Solutions, Cambridge, UK). For flow cytometric analysis, it was performed to detect the percentage of apoptosis cells. Briefly, 10^5^ cells were collected and stained with Annexin V-APC/7-ADD kit (Yeasen, Shanghai, China). Flow cytometry (Becton Dickinson) was used for quantifying the positive stained cells and then FlowJo-V10 software was used for analyzed the data.

### Coimmunoprecipitation (co-IP) and ubiquitination assay

After washing with phosphate buffer saline (PBS), cells were lysed in RIPA buffer and gentle rocked for 20 min at 4 °C. Lysates were centrifuged at 12,000 g for 15 min. The supernatant was collected and incubated with primary antibody pre-absorbed protein A and G sepharose beads at 4 °C for 6 h. Then, the sediments were washed with the RIPA buffer and boiled in 1x loading buffer for 10 min. Protein expression was detected by Western blot. For ubiquitination assays, A375-Vector and A375-TRIM44 cells were transfected with HA-Ubiquitin (HA-Ubi) and treated with MG132 (5 μmol, Selleck, China) for 8 h. Then, cells were used in Co-IP and Western blot assays for the detection of HA-Ubi expression.

### Bioinformatics prediction and luciferase reporter assay

GEPIA (Gene Expression Profiling Interactive Analysis, http://gepia.cancer-pku.cn/) was used to detect TRIM44 mRNA levels in different tumors. The miRNA target site within the 3′-UTR region of TRIM44 mRNA was predicted using TargetScan (http://www.targetscan.org/) and miRWalk (http://zmf.umm.uni-heidelberg.de/apps/zmf/mirwalk2/). Luciferase reporter assays were performed as previously reported [23]. Briefly, 293 T cells were transiently co-transfected with the luciferase reporter vector containing TRIM44–3′-UTR-wide type (WT) or TRIM44–3′-UTR-mutant (MUT) and either miR-26b-5p mimics or empty vectors. After 60 h, cells were collected and analyzed for Firefly and Renilla luciferase activity using the Dual-Luciferase Reporter Assay System (KeyGEN BioTECH, China).

### Statistical analysis

The results were analyzed using SPSS 20.0 software (Inc., Chicago, IL) and are presented as mean ± standard deviation (SD). All experiments were performed at least three times. Statistical *p* values were analyzed by the two-tailed Student’s *t* tests or one-way ANOVAs. Kaplan-Meier analyses and log-rank tests were used to analyze the overall survival and cumulative recurrence rate. A *p* < 0.05 was considered statistically significant.

## Results

### TRIM44 is overexpressed in melanoma and TRIM44 overexpression correlates with a poor prognosis

**Fig. 1:**
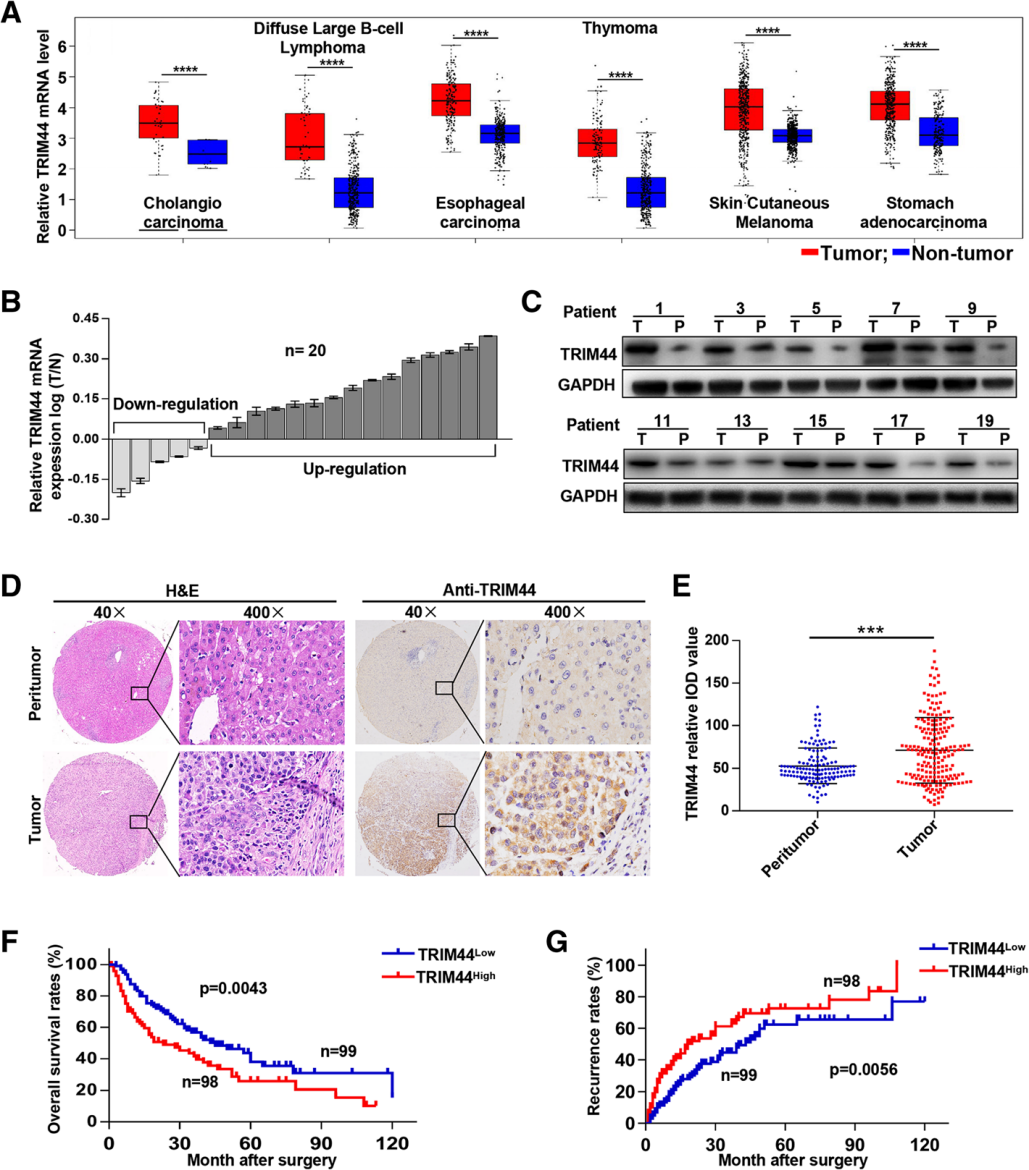
Overexpression of TRIM44 positively correlates with poor prognosis of melanoma patients. **a**, TRIM44 mRNA levels in different cancers were analyzed by GEPIA. **b**, TRIM44 mRNA levels in 20 matched pairs of melanoma and peritumoral tissues quantified by qRT-PCR. **c**, Representative bands of TRIM44 protein levels in 20 matched pairs of melanoma and peritumoral tissues. **d**, Representative images of TMA stained with H&E and immunohistochemistry for TRIM44. **e**, TRIM44 expression levels in melanoma and peritumoral tissues analyzed by relative IOD value. Overall survival and recurrence analyses of 197 melanoma patients according to TRIM44 expression. ***p < 0.001, ****p < 0.0001. T, tumor; P, peritumor

By retrieving the database, we found that TRIM44 is significantly upregulated in multiple cancers, including melanoma (Fig. 1a). Here, qRT-PCR and Western blot results indicated that TRIM44 expression was significantly upregulated in melanoma tissues relative to matched peritumorous tissues (Fig. 1b and c). Histochemical analyses found that TRIM44 was primarily expressed in the cytoplasm and there was significantly higher TRIM44 expression in melanoma samples compared with matched normal tissues (Fig. 1d and e). Patients with high levels of TRIM44 expression had lower overall survival (*p* = 0.0043) and higher recurrence rates (*p* = 0.0056) than those with low TRIM44 expression levels (Fig. 1f and g). Moreover, high levels of TRIM44 correlated with advanced Breslow depth (*p* = 0.025), distant metastasis (*p* = 0.012), and TNM stage (*p* = 0.002, Table 1). Importantly, univariate and multivariate analyses indicated that the TRIM44 level, Breslow depth, and TNM stage were independent indicators for melanoma patient prognoses (Table 2). These results demonstrate that TRIM44 is highly expressed in melanoma tissue, and that high expression is associated with a poor prognosis for these patients.

**Table 1:** Correlations Between TRIM44 with Clinicopathologic Features in 197 Melanoma Patients

Variable	Number of Patients		*P* value*
	TRIM44^low^	TRIM44^high^	
Age, year			
< 60	36	42	0.351
≥ 60	63	56	
Gender			
Male	49	56	0.282
Female	50	52	
Anatomic site			
Acra	48	53	0.395
Trunk	22	26	
Other	29	21	
Histologic type			
Superfical spreading	27	19	0.287
Nodular	23	18	
Acral	29	40	
Lentigo maligna	20	21	
Ulceration			
Present	15	11	0.416
Absent	84	87	
Breslow depth (mm)			
≤ 2	49	64	0.025
> 2	50	34	
Clark level			
I-III	54	53	0.948
IV-V	45	45	
Lymph nodes metastasis			
No	69	73	0.453
Yes	30	25	
Distant metastasis			
No	82	66	0.012
Yes	17	32	
Clinical stage			
I-II	73	51	0.002
III-IV	26	47	

Note: A chi-square test was used for comparing groups between low and high TRIM44 expression. *, *p* < 0.05 was considered significant

**Table 2:** Univariate and Multivariate Analyses of Factors Associated With survival and recurrence

	Overall survival				Cumulative recurrence			
	Multivariate Analysis				Multivariate Analysis			
Variable	Univariate P	HR	95%CI	P*	Univariate P	HR	95%CI	P
Age, year (≥60 vs. <60)	0.230			NA	0.286			NA
Gender (Men vs. Women)	0.741			NA	0.798			NA
Anatomic site (Acra vs. Trunk vs. Other)	0.858			NA	0.887			NA
Histologic type (Superfical spreading vs. Nodular vs. Acral vs. Lentigo maligna)	0.089			NA	0.054			NA
Ulceration (Present vs. Absent)	0.284			NA	0.249			NA
Breslow depth (mm) (≤2 vs. > 2)	0.014	1.839	1.347–2.916	0.037	0.024	1.526	1.135–2.527	0.046
Clark level (I-III vs. IV-V)	0.023			NS	0.031			NS
Lymph nodes metastasis (Yes vs. No)	0.015			NS	0.015			NS
Distant metastasis (Yes vs. No)	0.002			NS	0.003			NS
Clinical stage (Yes vs. No)	0.004	3.853	2.325–6.518	0.001	< 0.001	4.104	2.395–6.718	< 0.001
TRIM44 staining (Low vs. High)	0.010	1.297	1.040–2.215	0.019	0.005	1.230	1.012–2.010	0.004

Note: *OS* overall survival, *DFS* disease free survival, *NS* not significant, *NA* not adopt

*, *p* < 0.05 was regarded as statistically significant, *p* value was calculated using Cox proportional hazards regression

### Elevated TRIM44 levels promote melanoma progression in vivo and in vitro

**Fig. 2:**
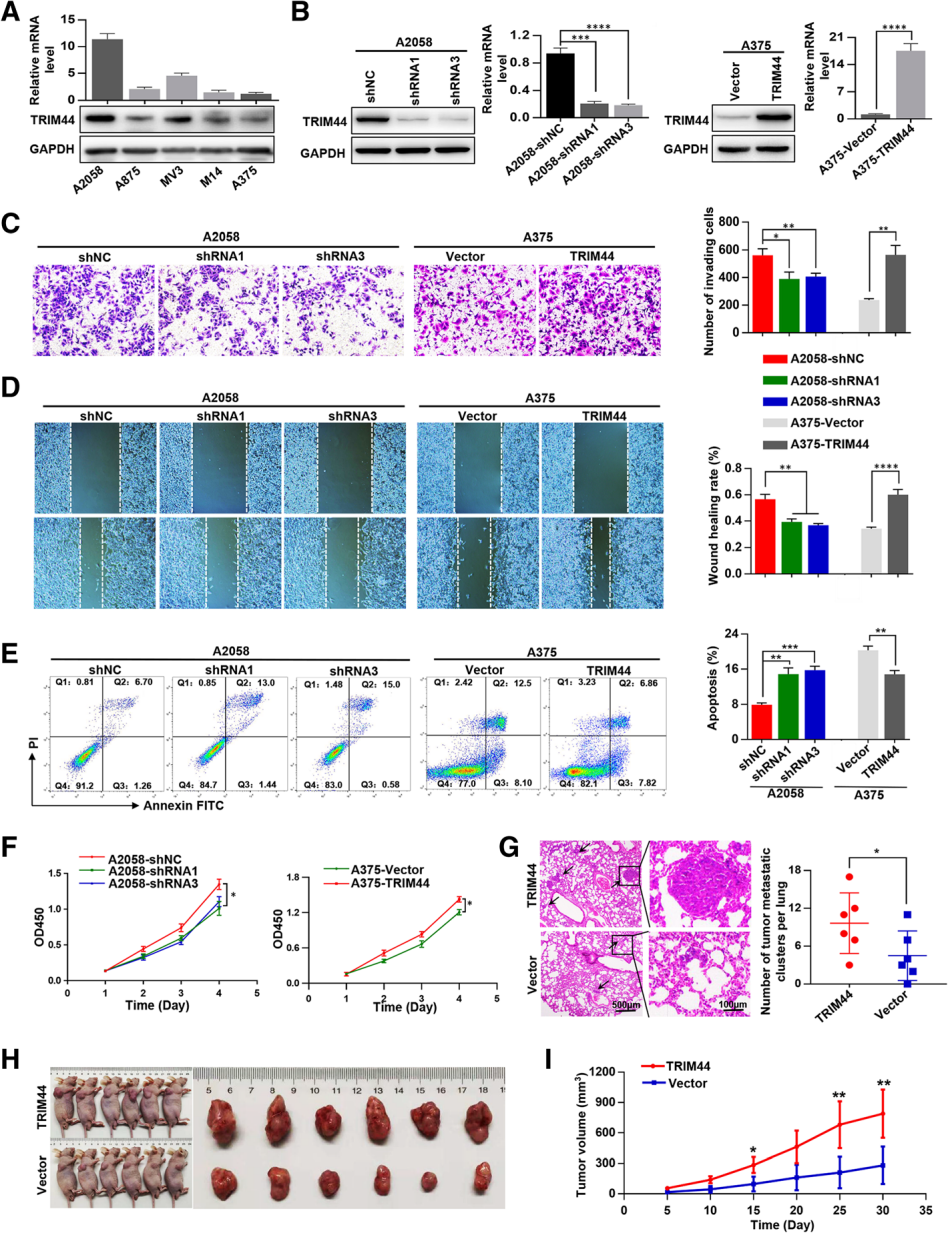
Elevated TRIM44 promotes melanoma cell progression both in vitro and in vivo. **a**, mRNA and protein levels of A2058, A875, MV3, M14, A375 cells. **b**, A2058 cells were transfected with short hairpin RNAs (shRNAs), while A375 cells were transfected with TRIM44. Transfection efficiencies were assessed by Western blot and qRT-PCR. **c**, Invasive abilities conveyed by A2058-shNC, A2058-shRNAs, A375-Vector, A375-TRIM44 were measured by Matrigel invasion assays. **d**, Migration ability conveyed by A2058-shNC, A2058-shRNAs, A375-Vector, A375-TRIM44 were assessed by wound-healing migration assays. **e**, Flow cytometric assay was used for detecting the apoptosis cells of A2058-shNC, A2058-shRNAs, A375-Vector, A375-TRIM44. **f**, Cell proliferation in A2058-shNC, A2058-shRNAs, A375-Vector, A375-TRIM44 cells was detected by CCK-8 assays. **g**, The in vivo effects of TRIM44 forced expression on lung metastasis were investigated by tail vein injection, and the number of metastases was examined by H&E staining. **h**, A375-TRIM44 and A375-Vector cells were subcutaneously inoculated in nude mice to test the proliferative ability in vivo. **i**, Tumor growth curves of the subcutaneous xenografts. ^*^p < 0.05, ^**^p < 0.01, ^***^p < 0.001, ^****^p < 0.0001

First, TRIM44 expression was detected in five melanoma cell lines (Fig. 2a). We transfected TRIM44 shRNAs into A2058 cells for its high TRIM44 expression. TRIM44 cDNA vectors were transfected into A375 cells for its low TRIM44 expression. Transfection efficiencies were verified by Western blot and qRT-PCR **(**Fig. 2b**).** We found that downregulation of TRIM44 inhibited the invasion, migration, apoptosis resistance and proliferation of A2058 cells, while upregulation of TRIM44 significantly enhanced these functions in A375 cells (Fig. 2c-f). In the pulmonary metastasis model, the incidence of lung metastasis for the A375-TRIM44 group was higher than that of A375-Vector group **(**Fig. 2g). In the subcutaneous xenograft model, growth curves indicated that tumors in the A375-TRIM44 group had higher proliferative ability than tumors in the A375-Vector group (Fig. 2h and i). These results imply that upregulation of TRIM44 promotes melanoma progression both in vivo and in vitro.

### TRIM44 regulates melanoma migration and invasion via EMT

**Fig. 3:**
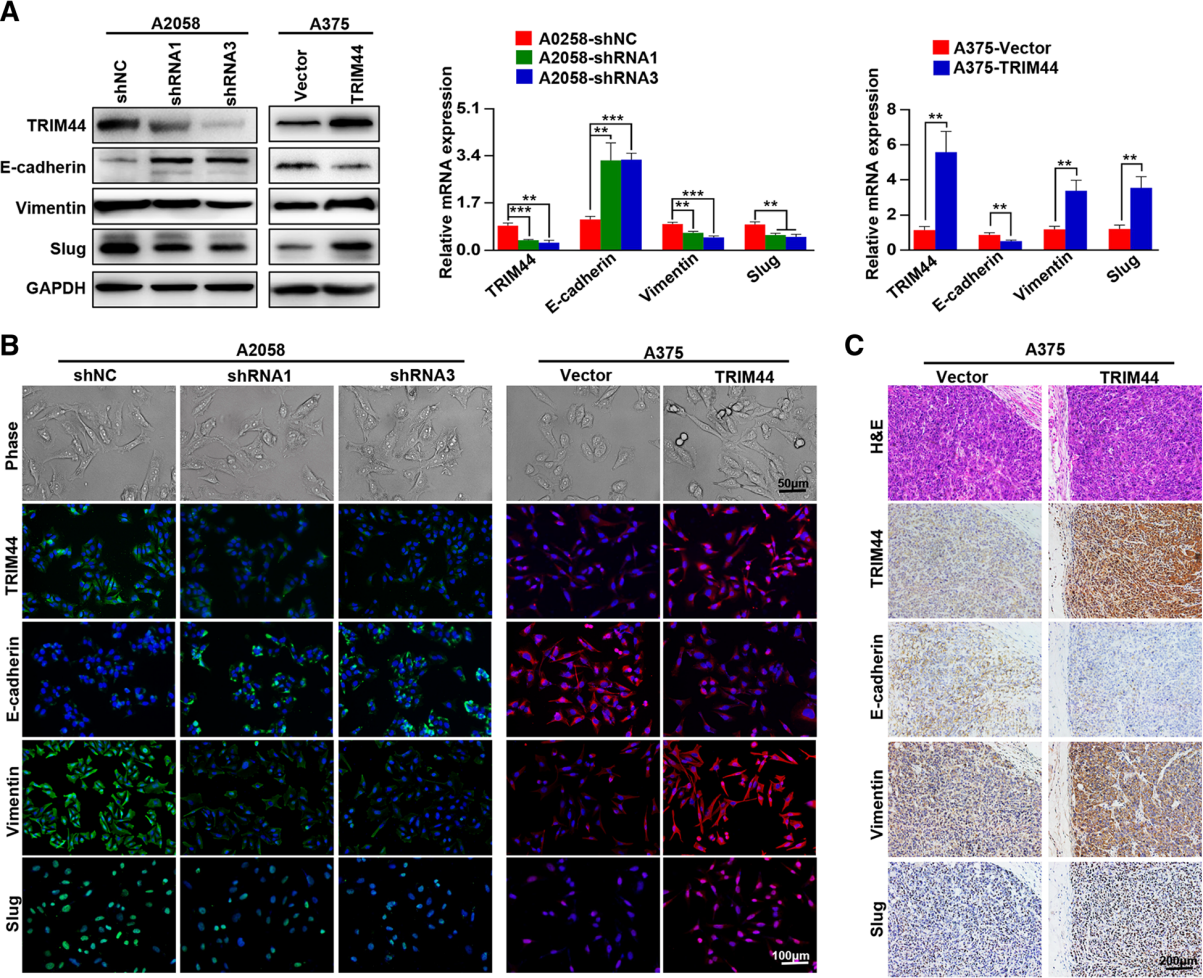
Overexpression of TRIM44 promotes cellular EMT in melanoma. **a**, Western blot (left) and qRT-PCR (right) analyses were used to detect the expression levels of E-cadherin, Vimentin, and Slug in the indicated cells. **b**, Cellular morphologies of the indicated cells (upper) and expression of TRIM44, E-cadherin, Vimentin, and Slug were analyzed by immunofluorescence staining (lower). **c**, Expression of TRIM44, E-cadherin, Vimentin, and Slug was analyzed by IHC. ^**^p < 0.01, ^***^p < 0.001

Previous studies have shown that high levels of TRIM44 induce cellular EMT in hepatocellular carcinoma (HCC) and non-small cell lung carcinoma (NSCLC) [24, 25]. Here, we show that downregulation of TRIM44 was associated with high levels of E-cadherin expression and low levels of Vimentin and Slug. Conversely, TRIM44 upregulation inhibited E-cadherin expression and upregulated Vimentin and Slug expression (Fig. 3a). We found that both A2058-shRNAs and A375-Vector cells took on the cobblestone-like appearances typical of normal epithelial cells, while both A2058-shNC and A375-TRIM44 developed the spindle-like morphology of fibroblasts (Fig. 3b, upper). This cell morphology indicated that cells with high levels of TRIM44 develop an EMT-like phenotype, finding that was supported by immunofluorescence (Fig. 3b, lower) and immunohistochemistry analyses of subcutaneous xenograft melanoma tissues (Fig. 3c). Taken together, these results indicate that TRIM44 induces EMT, and thereby contributes to melanoma progression.

### High levels of TRIM44 induce EMT by activating the AKT/mTOR pathway

**Fig. 4:**
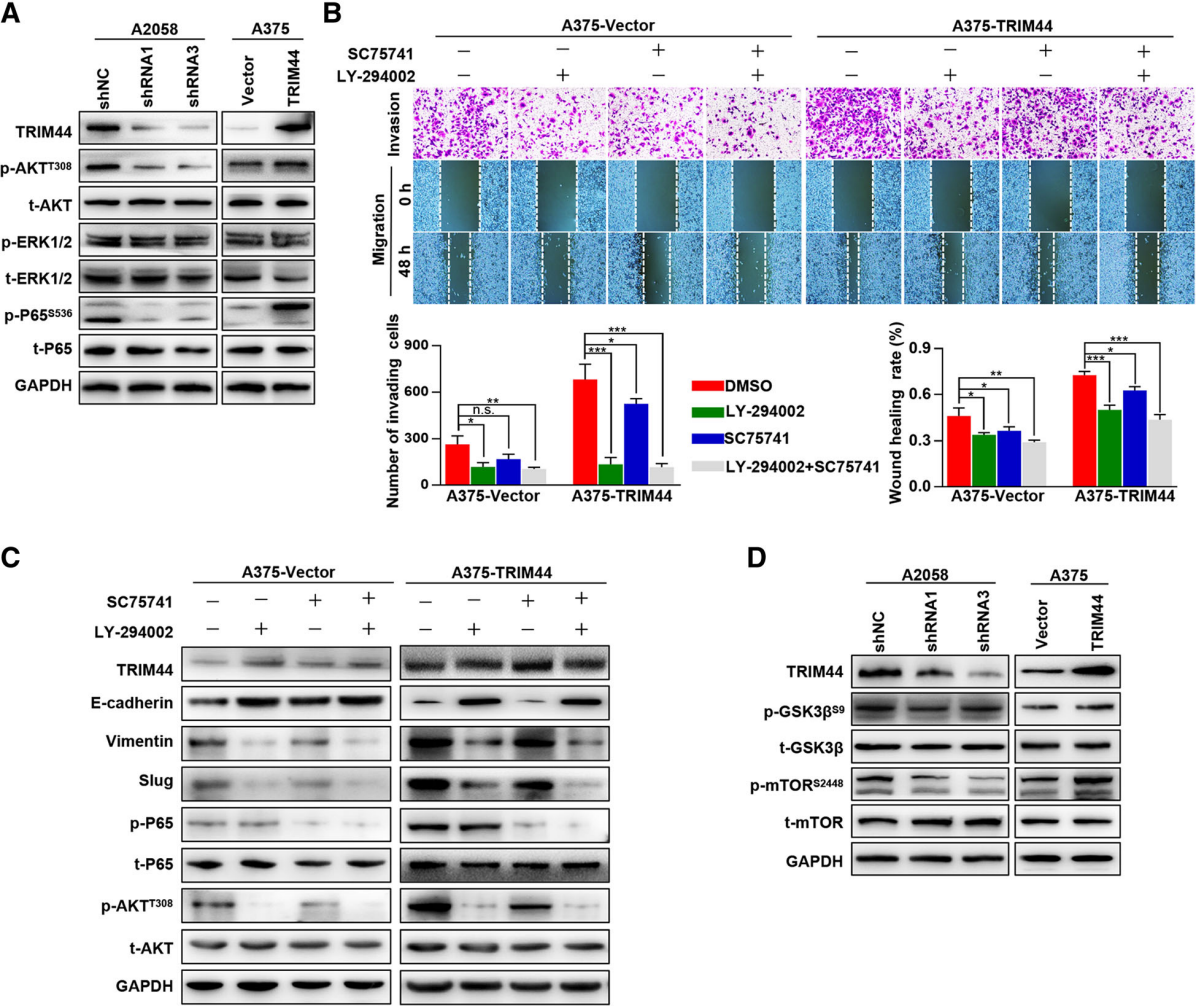
Elevated TRIM44 facilitates cellular EMT in melanoma via the AKT/mTOR pathway. **a**, Western blot was used to detect levels of p-AKT, AKT, p-ERK1/2, ERK1/2, p-P65, and P65 in the indicated cells. **b**, Invasion and migration assays were performed in A375-Vector and A375-TRIM44 cells after incubation with either LY-294002 (AKT pathway inhibitor), SC75741 (NF-κB pathway inhibitor), or both. **c**, Western blot was used to detect levels of TRIM44, EMT markers, p-P65, P65, p-AKT, and AKT in A375-Vector and A375-TRIM44 cells after incubation with LY-294002, SC75741, or both. **d**, Western blot was used to detect the levels of TRIM44, p-GSK3β, GSK3β, p-mTOR, and mTOR in A2058-shNC, A2058-shRNAs, A375-Vector, and A375-TRIM44 cells. ^**^p < 0.01, ^***^p < 0.001, ^****^p < 0.0001

Multiple pathways are involved in TRIM44-induced tumor progression, including AKT, ERK1/2, and NF-κB signaling [24–26]. We found that upregulation of TRIM44 activated the AKT and NF-κB pathways, while downregulation of TRIM44 inactivated the AKT and NF-κB pathways. There was no variation of p-ERK1/2 levels (Fig. 4a). Through pathway inhibitors, we found that TRIM44-induced invasion, migration, and EMT were reversed by LY-294002 (AKT inhibitor, 50 μM, 24 h), while not by SC75741 (NF-κB inhibitor, 5 μM, 24 h, Fig. 4b and c). Furthermore, we found that high levels of TRIM44 could upregulate levels of p-mTOR (S2448) (Fig. 4d). Taken together, these data indicate that upregulation of TRIM44 activates the AKT/mTOR pathway, which in turn promotes melanoma cell EMT.

### TRIM44 activates AKT/mTOR pathway by protecting TLR4 from ubiquitin

**Fig. 5:**
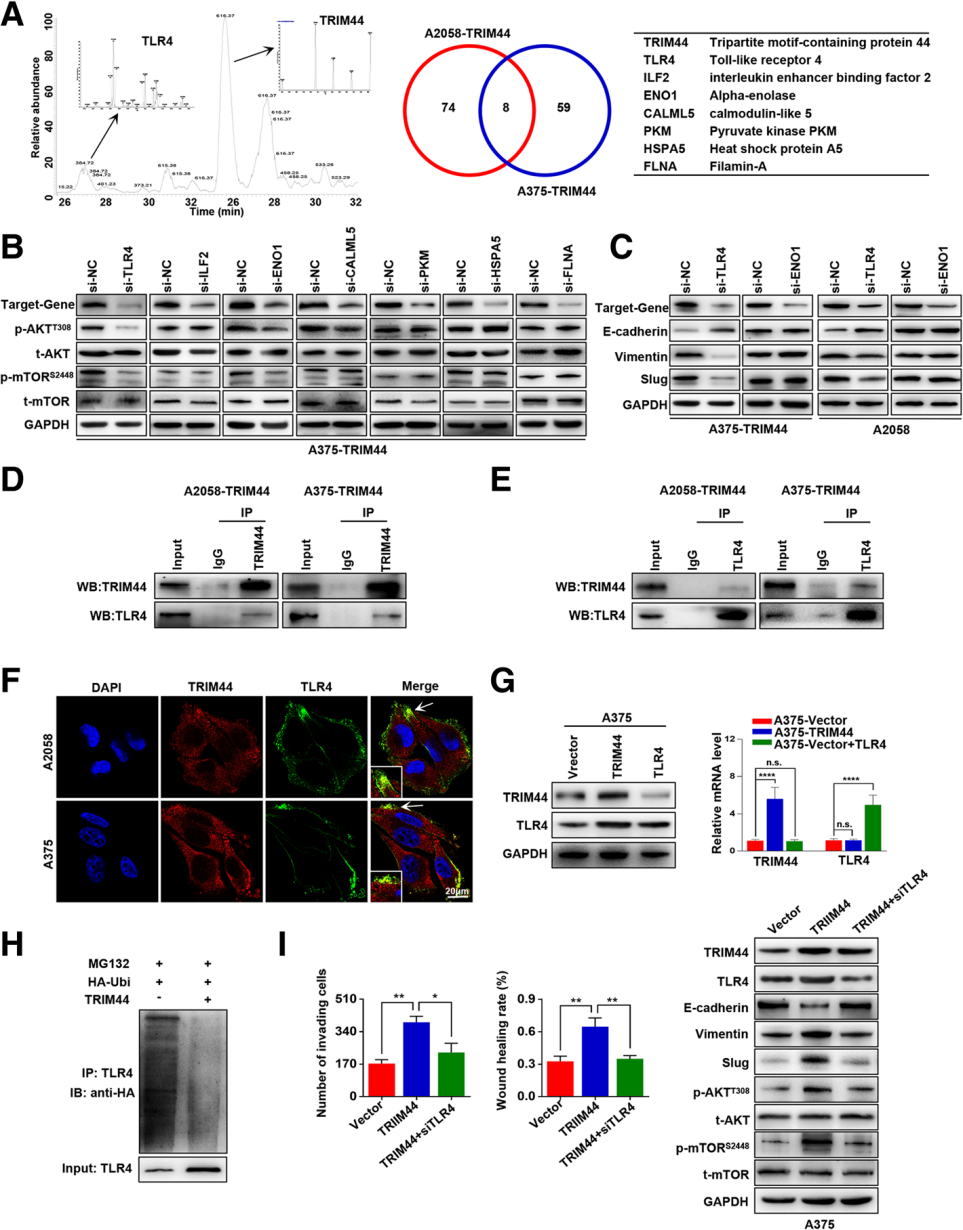
TRIM44 activates the AKT/mTOR pathway by blocking ubiquitination of TLR4. **a**, The binding partners of TRIM44 were analyzed by a combination of Co-IP and mass spectrometry, and seven overlapping proteins are presented. **b**, Western blot was used to quantify levels of p-AKT, AKT, p-mTOR and mTOR when the expression of relevant genes was inhibited. The AKT/mTOR pathway was affected by TLR4 and ENO1 interference. **c**, Western blot was used to detect levels of E-cadherin, Vimentin, and Slug in the indicated cells. Only TLR4 altered the expression of EMT markers. **d** and **e**, Co-IP used to verify formation of the TRIM44/TLR4 complex. **f**, Immunofluorescence used to verify formation of the TRIM44/TLR4 complex. **g**, Western blot and qRT-PCR used to quantify expression of TRIM44 and TLR4 in the indicated cells. TRIM44 overexpression led to upregulation of TLR4 proteins, but did not influence mRNA levels. **h**, Ubiquitination assay for the effects of TRIM44 on TLR4 ubiquitination. HA-Ubi were co-transfected into A375-Vector and A375-TRIM44 cells. **i**, Invasion and migration assays were performed in the indicated cells (left); Western blot was used to quantify expression levels of TRIM44, TLR4, EMT markers, p-AKT, AKT, p-mTOR^s2448^ and mTOR in the indicated cells (right). After transfecting siTLR4 into A375-TRIM44 cells, TRIM44-induced EMT was significantly attenuated. n.s., not significant, ^*^p < 0.05, ^**^p < 0.01, ^***^p < 0.001

Based on previous reports that TRIM44 functions as a deubiquitinase [9, 10], we investigated the TRIM44 functional substrate. A combination of co-IP and mass spectrometric analyses identified seven overlapping proteins in both A375-TRIM44 and A2058-TRIM44 cells (Fig. 5a). To determine whether these proteins are involved in TRIM44 induced AKT/mTOR activation, we individually assayed RNAi candidates for interference in expression of these proteins. The findings showed that TLR4 and ENO1 interfered with the AKT/mTOR pathway (Fig. 5b). We then determined that decreased expression of TLR4, but not ENO1, upregulated E-cadherin and downregulated both Vimentin and Slug (Fig. 5c). Co-IP and immunofluorescence assays verified the overlap between TRIM44 and TLR4 proteins (Fig. 5d-f). Furthermore, we found that overexpression of TRIM44 significantly upregulated the TLR4 protein, whereas overexpression of TLR4 did not influence the expression of TRIM44. This suggests that TLR4 is the substrate of TRIM44. In addition, TRIM44 influenced TLR4 expression at the protein level, but not the mRNA level (Fig. G). Ubiquitination assays showed that overexpression of TRIM44 decreased TLR4 protein polyubiquitination (Fig. 5h). Then, we transfected siTLR4 into A375-TRIM44 cells, which significantly attenuated the invasion, migration, and EMT properties induced by TRIM44 (Fig. 5i). Together, these results demonstrate that TRIM44 deubiquitinates and stabilizes TLR4 to activate the AKT/mTOR pathway.

### MiR-26b-5p represses TRIM44 expression by directly targeting its 3′-UTR

**Fig. 6:**
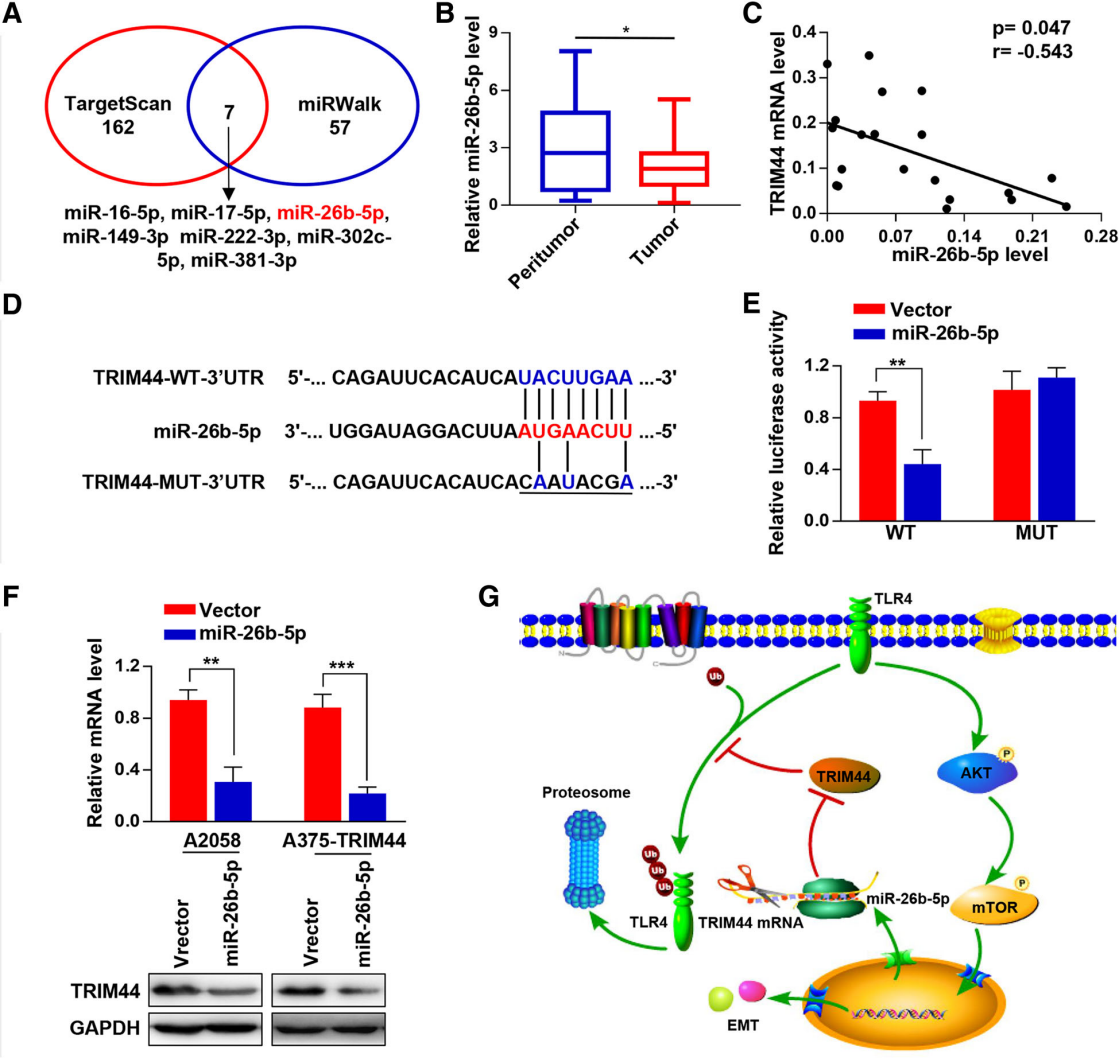
MiR-26b-5p represses TRIM44 expression by directly targeting its 3′-UTR. **a**, Two independent databases were searched to computational predict miRNAs that targeting the 3′-UTR of TRIM44. **b**, qRT-PCR was used to quantify miR-26b-5p expression levels in 20 matched pairs of melanoma and paratumoral tissues. **c**, Correlation analyses between TRIM44 mRNA and miR-26b-5p were performed using the Spearman correlation coefficient. TRIM44 mRNA negatively correlated with miR-26b-5p expression. **d**, The putative miR-26b-5p binding sequence in the 3′-UTR of TRIM44. **e**, 293 T cells were co-transfected with WT or MUT luciferase reporter plasmids and miR-26b-5p mimics or negative control. Luciferase activity was evaluated 48 h post-transfection. **f**, qRT-PCR and Western blot were used to quantify TRIM44 expression in the indicated cells. Overexpression of miR-26b-5p in A2058 and A375 cells significantly inhibited the expression of TRIM44 a both the mRNA and protein levels. **g**, A working model depicting the miR-26b-5p-TRIM44-TLR4-AKT-mTOR axis. ^*^p < 0.05, ^**^p < 0.01, ^***^p < 0.001

To explore the upstream regulators of TRIM44, we performed TargetScan and miRWalk to detect miRNAs that potentially target TRIM44 (Fig. 6a). Among the identified overlapping miRNA, we focused on miR-26b-5p based on its known role in EMT inhibition. We found that miR-26b-5p was downregulated in the melanoma tissue samples compared with paired paratumoral tissue samples (Fig. 6b), and that there was a negatively relationship between the expression of TRIM44 mRNA and miR-26b-5p (*p* = − 0.047. r = − 0.543, Fig. 6c). Luciferase reporter assays showed that expression of miR-26b-5p significantly inhibited the activity of luciferase that carried wild type, but not the mutant 3′-UTR of TRIM44. This indicates that miR-26b-5p may suppress TRIM44 expression through its 3′-UTR binding sequence (Fig. 6d and e). Moreover, we found that overexpression of miR-26b-5p in A2058 and A375 cells significantly inhibited the expression of TRIM44 at both the mRNA and protein levels (Fig. 6 f). These results strongly support the hypothesis that TRIM44 is a direct target of miR-26b-5p.

### Overexpression of TRIM44 and TLR4 predicts poorer prognosis in melanoma patients

**Fig. 7:**
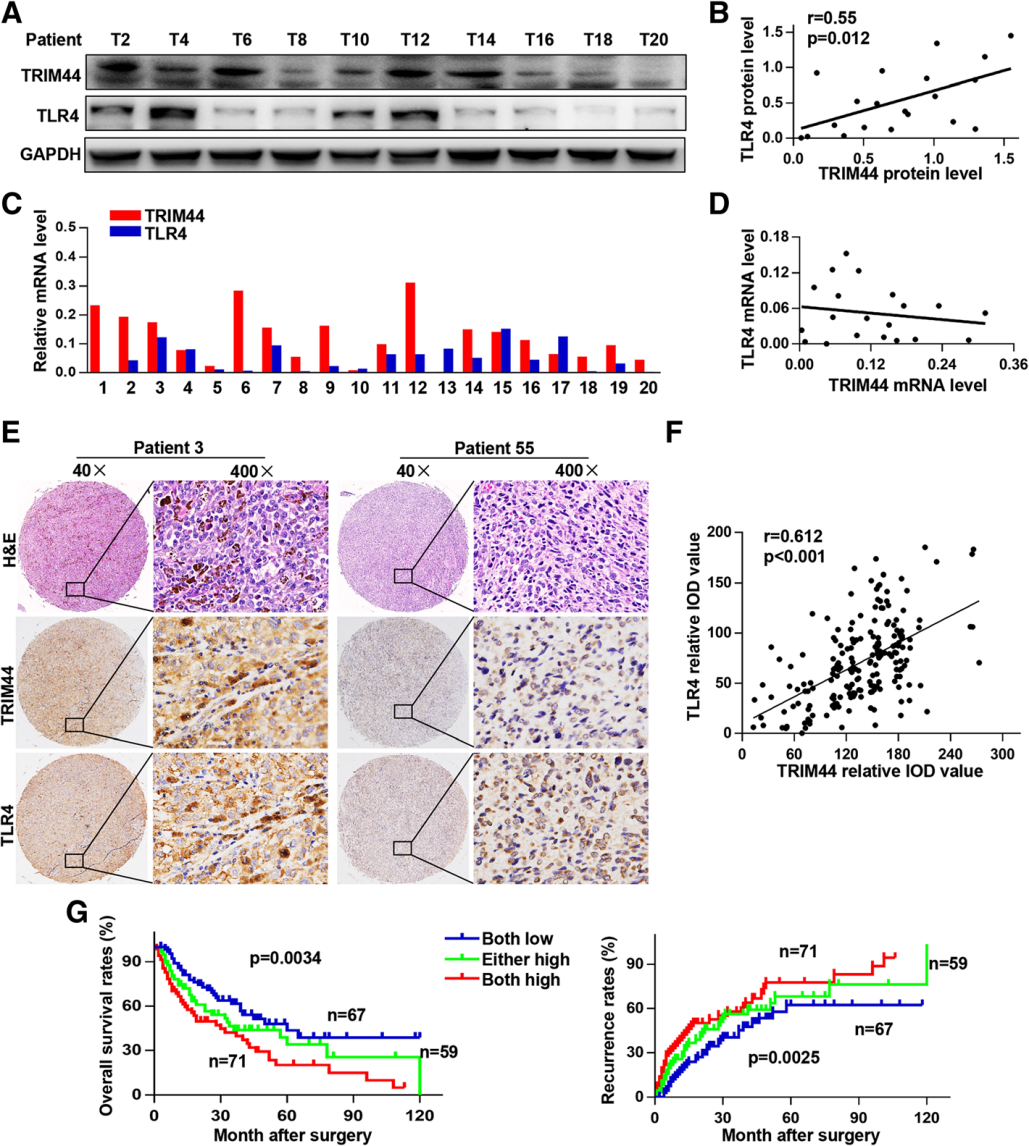
Overexpression of TRIM44 and TLR4 is an indicator for poor prognosis in melanoma patients. **a** and **b**, Representative bands of TRIM44 and TLR4 protein levels in 20 melanoma tissues are shown. Analyses of the correlation between TRIM44 and TLR4 was performed using the Spearman correlation coefficient. **c** and **d**, TRIM44 and TLR4 mRNA levels in 20 melanoma tissues. Analysis of the correlation between TRIM44 and TLR4 was performed using the Spearman correlation coefficient. There was a positive relationship between TRIM44 and TLR4 at the protein level, but not the mRNA level. **e**, Representative images of TMA stained with H&E, immunohistochemistry for TRIM44 and TLR4. **f**, Correlation analysis of the relative IOD values of TRIM44 and TLR4 using the Spearman correlation coefficient. TRIM44 positively correlated with TLR4 at the protein level. **g**, Kaplan-Meier analyses showing relationships between differential expression of TRIM44 and TLR4 and either overall survival or recurrence using the log-rank test. High levels of TRIM44 and TLR4 were associated with shortened overall survival and higher recurrence rates. ^*^ *p* < 0.05, ^***^p < 0.001. T, tumor; P, peritumor

To further explore the relationship between TRIM44 and TLR4, we quantified TRIM44 and TLR4 expression in 20 melanoma tissue samples. We identified a positive relationship between TRIM44 and TLR4 at the protein level (r = 0.55, *p* = 0.012, Fig. 7a, b), but not the mRNA level (Fig. 7c, d). Immunohistochemical analyses further supported findings of a positive correlation between TRIM44 and TLR4 (r = 0.612, *p* < 0.001, Fig. 7e, f). In addition, we found that patients with high levels of trim44 and TLR4 had reduced overall survival and higher recurrence rates than other patients (Fig. 7g). This suggests that overexpression of TRIM44 and TLR4 are indicators of a poor prognosis in melanoma patients.

## Discussion

The present study demonstrates that TRIM44 is significantly amplified in melanoma tissues, and overexpression of TRIM44 is associated with a malignant phenotype of melanoma. Our findings indicate that, in terms of mechanism, TRIM44 deubiquitinates and stabilizes TLR4, which activates the AKT/mTOR pathway and induces cell EMT. Moreover, TRIM44 is a downstream target gene of miR-26b-5p, which acts as a TRIM44 suppressor (Fig. 6g). Clinically, we find that high levels of TRIM44 combined with upregulation of TLR4 serves as a promising prognostic indicator in melanoma patients.

Several studies report that TRIM44 plays a crucial role in the progression of human cancers. For example, Kawabata et al. demonstrated that high levels of TRIM44 promote cell proliferation and migration by enhancing NF-κB signaling in breast cancer [26]. Peng et al. found that elevated TRIM44 facilitates cellular EMT via MAPK pathway in intrahepatic cholangiocarcinoma (ICC) [24]. Our present study is the first to show that TRIM44 is significantly upregulated in melanoma tissues using a large number of clinical samples, a result that is consistent with that of the public database. Moreover, we found that elevated TRIM44 promoted cell proliferation, invasion, migration and apoptosis resistance in vitro, and promoted melanoma growth and metastasis in vivo. Importantly, high levels of TRIM44 induced melanoma cell EMT, which is one of the most important mechanisms for the promotion of tumor metastasis. Retrospective review of the literature indicates that TRIM44 triggers multiple classical pathways, including AKT/mTOR pathway. For example, Xiong et al. showed that elevated TRIM44 expression promotes human esophageal cancer development via the AKT/mTOR pathway [25]. Xing et al. reports that TRIM44 contributed to EMT and cell cycle progression by modulating the AKT/mTOR pathway, thereby stimulating lung cancer cell metastasis and proliferation [27]. In line with these studies, we found that upregulation of TRIM44 activates AKT/mTOR signaling. Furthermore, EMT induced by TRIM44 can be reversed by a AKT inhibitor. Unlike other members of the TRIM protein family, TRIM44 contains a zinc finger UBP in the N-terminal, instead of a RING domain, and therefore likely acts as a deubiquitinating enzyme [9, 10]. Our study supports this, as we found that upregulation of TRIM44 inhibited polyubiquitination of the substrate protein and maintained its stability. To identify the TRIM44 substrate, we performed mass spectrometry and found seven overlapping proteins. Among them, only knock-down of TLR4 expression decreased p-AKT, p-mTOR levels, and inhibited cell EMT. We therefore conclude that high levels of TRIM44 activate AKT/mTOR signaling by combining with TLR4 and stabilizing its expression. Subsequent Co-IP and immunofluorescence assays confirmed that there is a direct interaction between TRIM44 and TLR4 proteins. In combination with the ubiquitin assay findings, this provides strong evidence that TRIM44 deubiquitinates TLR4 to activate AKT/mTOR signaling. Of course, this post-translational modification may not limit to melanoma, as the TRIM44-AKT-mTOR axis is common in multiple tumors. We also showed that miR-26b-5p regulates TRIM44 expression. Corresponding with this, miR-26b-5p is reported to be downregulation in melanoma [28] and has been found to suppress cellular EMT, invasion, and metastasis in hepatocellular carcinoma [29]. Hence, downregulation of miR-26b-5p is one mechanism for the upregulation of TRIM44.

## Conclusions

Our findings describe a miR-26b-5p-TRIM44-TLR4-AKT-mTOR axis in melanoma cells, and provides new insights into the mechanisms underlying melanoma progression. Importantly, TRIM44 may be a useful prognostic indicator for melanoma and provides a potential new target for melanoma therapy.

## Additional files

Additional file 1: **Table S1.** List of Primary Antibodies Used In the study. (DOCX 15 kb)

Additional file 2: **Table S2.** Sequences of Primer for Real-time Polymerase Chain Reaction. (DOCX 13 kb)

## Abbreviations

CCK-8: Cell Counting Kit-8
ECL: Electrogenerated chemiluminescence
EMT: Epithelial-mesenchymal transition
GSEA: Gene set enrichment analysis
H&E: Hematoxylin and eosin
HA: Hemagglutinin
HCC: Hepatocellular carcinoma
IHC: Immunohistochemistry
IP: Immunoprecipitation
miRNAs: MicroRNAs
mRNAs: Messenger RNAs
MS: Mass spectrometry
NSCLC: Non-small cell lung carcinoma
PBS: Phosphate buffer saline
PVDF: Polyvinylidene difluoride
qRT-PCR: Quantitative real-time polymerase chain reaction
SD: Standard deviation
SDS-PAGE: Sodium dodecyl sulfate-polyacrylamide gel electrophoresis
TLR4: TOLL-like receptor 4
TMA: Tissue microarray
TRIM44: Tripartite motif-containing protein 44
UBP: Ubiquitin protease domain
UTR: Untranslated region
WT: Wide type
