# Supplementary material for: TRIM44 activates the AKT/mTOR signal pathway to induce melanoma progression by stabilizing TLR4
Source: J Exp Clin Cancer Res. 2019 Mar 28;38:137. doi: 10.1186/s13046-019-1138-7 (PMC6437891; doi:10.1186/s13046-019-1138-7)
Supplement: Supplementary file 1 — Table S1. List of Primary Antibodies Used In the study. (DOCX 15 kb) [file 13046_2019_1138_MOESM1_ESM.docx]

**Supplementary Table I List of Primary Antibodies Used In the study**

| Antibody | Applications | Company |
| --- | --- | --- |
| TRIM44 | WB, IF | Proteintech (66249-1-Ig) |
| TRIM44 | WB, IHC | Proteintech (11511-1-AP) |
| GAPDH | WB, IF, IHC | Abcam (ab8245) |
| E-cadherin | WB, F, IF,IHC | CST (3195) |
| Vimentin | WB, F, IF,IHC | CST (5741) |
| Slug | WB, IP, IF, F | CST (9585) |
| p-AKT^T308^ | WB, IHC | Abcam (ab38449) |
| AKT | WB, IHC, ELISA, | Abcam (ab8805) |
| p-ERK | WB, IP, IHC, IF, F | CST (4370) |
| ERK | WB, IP, IHC, IF, F | CST (4695) |
| p-P65^S536^ | WB, IP, IF, F, | CST (3033) |
| P65 | WB, IP, IF, F, ChIP | CST (8242) |
| p-GSK3β^S9^ | WB, IP, IF | Abcam (ab131097) |
| GSK3β | WB, IF, IHC, F | Abcam (ab3239) |
| p-mTOR^S2448^ | WB, IF, ELISA | CST (5536) |
| mTOR | WB, IHC, IF, F | CST (2983) |
| TLR4 | WB, IP, IHC, F, ELISA | Abcam (ab13556) |
| ILF2 | WB, IHC, ICC | Abcam (ab28772) |
| ENO1 | WB, IHC, ICC | Abcam (ab85086) |
| CALML5 | WB, IHC, ICC | Abcam (ab122665) |
| PKM | WB, IHC, ICC | Abcam (ab38237) |
| HSPA5 | WB, IHC, ICC, IP | Abcam (ab21685) |
| FLNA | WB, IHC, ICC, ELISA | Abcam (ab51217) |
| HA | WB, IP, IF, ELISA | Proteintech (51064-2-AP) |

**Abbreviations:** WB, western blot; IHC, immunohistochemistry; IF, immunofluorescence; IP, immunoprecipitation; ELISA, enzyme linked immunosorbent assay; F, Flow cytometric analysis; ChIP, Chromatin immunoprecipitations.
