# Supplementary material for: TRIM44 activates the AKT/mTOR signal pathway to induce melanoma progression by stabilizing TLR4
Source: J Exp Clin Cancer Res. 2019 Mar 28;38:137. doi: 10.1186/s13046-019-1138-7 (PMC6437891; doi:10.1186/s13046-019-1138-7)
Supplement: Supplementary file 2 — Table S2. Sequences of Primer for Real-time Polymerase Chain Reaction. (DOCX 13 kb) [file 13046_2019_1138_MOESM2_ESM.docx]

**Supplementary Table II Sequences of Primer for Real-time Polymerase Chain Reaction**

| **TRIM44** |  |
| --- | --- |
| Forward | 5’-AGGCAGCTCATCTGTGTCCT-3’ |
| Reverse | 5’-GCCTTCAGTCCACCTGAGTC-3’ |
| **E-cadherin** |  |
| Forward | 5’-ATTTTTCCCTCGACACCCGAT-3’ |
| Reverse | 5’-TCCCAGGCGTAGACCAAGA-3’ |
| **Vimentin** |  |
| Forward | 5’-AGTCCACTGAGTACCGGAGAC-3’ |
| Reverse | 5’-CATTTCACGCATCTGGCGTTC-3’ |
| **Slug** |  |
| Forward | 5’-CGAACTGGACACACATACAGTG-3’ |
| Reverse | 5’-CTGAGGATCTCTGGTTGTGGT-3’ |
| **TLR4** |  |
| Forward | 5’-AGACCTGTCCCTGAACCCTAT-3’ |
| Reverse | 5’-CGATGGACTTCTAAACCAGCCA-3’ |
| **GAPDH** |  |
| Forward | 5’-GGTATGACAACGAATTTGGC-3’ |
| Reverse | 5’-GAGCACAGGGTACTTTATTG-3’ |
